# Clinique Medicale de Pavie

**Published:** 1833-05

**Authors:** 


### CLINIQUE MEDICALE DE PAVIE.

### *Itch successfully treated by Chlorate of Lime.*

By Professor Fantonelli.

The professor states that, after having employed the above remedy with considerable success in his private practice, he determined to try its effects upon a more extended scale in the hospital. It was employed in eight cases of itch, which were admitted about the same time; six men and two women. The latter, in whom the whole surface of the body was covered with the eruption, were cured in six days. Of the six men, five were completely cured from the fifth to the eighth day; in one instance, a young man, sixteen years old, the eruption of itch was followed, while he was treated in the same manner, by general eczema, which was however speedily relieved by warm baths. The original disease then returned, and was cured by fumigations of sulphur.

The strength of the lotion employed for adults was from one ounce and a half to two ounces of the chlorate in a pint of common water, and with this solution the parts affected were rubbed three or four times a-day. For infants, an ounce of the chlorate in the same quantity of water. Every three days the patient should be put into a warm bath, in order to remove from the skin the deposition of lime that may have formed upon it, and to relieve the irritation sometimes produced by the remedy, when it is used too strong, or when it is too frequently applied. It rarely happens that by this means the itch is not cured in eight days; and the professor feels confident that it is the most certain, the most prompt, and the most economical, treatment that has hitherto been adopted.—[*Ann. Universali di Med.*]

### COLLECTANEA.

**Floriferis ut apes in saltibus omnia libant,**
**Omnia nos, itidem, depascimur aurea dicta.**

### PHYSIOLOGY.

*On the Umbilical Vesicle.* By M. Mayer.

